# Effects of Moderate Consumption of Red Wine on Hepcidin Levels in Patients with Type 2 Diabetes Mellitus

**DOI:** 10.3390/foods11131881

**Published:** 2022-06-25

**Authors:** Jurica Nazlić, Diana Jurić, Ivana Mudnić, Zvonimir Boban, Ana Marija Dželalija, Leida Tandara, Daniela Šupe-Domić, Katarina Gugo, Mladen Boban

**Affiliations:** 1 Department of Intensive Medicine and Clinical Pharmacology, University Hospital of Split, Šoltanska 1, 21000 Split, Croatia; jnazlic@gmail.com; 2 Department of Pharmacology, University of Split School of Medicine, Šoltanska 2, 21000 Split, Croatia; ivana.mudnic@mefst.hr (I.M.); ana.marija.dzelalija@mefst.hr (A.M.D.); mladen.boban@mefst.hr (M.B.); 3 Department of Medical Physics and Biophysics, University of Split School of Medicine, Šoltanska 2, 21000 Split, Croatia; zvonimir.boban@mefst.hr; 4 Department of Medical Laboratory Diagnostics, University Hospital of Split, Šoltanska 1, 21000 Split, Croatia; leida.tandara@gmail.com (L.T.); daniela.supe.domic@ozs.unist.hr (D.Š.-D.); katarina.gugo@gmail.com (K.G.); 5 Department of Medical Chemistry and Biochemistry, University of Split School of Medicine, Šoltanska 2, 21000 Split, Croatia; 6 University Department of Health Studies, University of Split, Ruđera Boškovića 35, 21000 Split, Croatia

**Keywords:** type 2 diabetes, red wine, alternative-complementary therapy, iron, hepcidin, serum ferritin

## Abstract

Iron overload is often associated with type 2 diabetes (T2D), indicating that hepcidin, the master regulator of iron homeostasis, might be involved in diabetes pathogenesis. Alcohol consumption may also result in increased body iron stores. However, the moderate consumption of wine with meals might be beneficial in T2D. This effect has been mainly attributed to both the ethanol and the polyphenolic compounds in wine. Therefore, we examined the effects of red wine on hepcidin in T2D patients and non-diabetic controls. The diabetic patients (*n* = 18) and age- and BMI-matched apparently healthy controls (*n* = 13) were men, aged 40–65 years, non-smoking, with BMI < 35 kg/m^2^. Following a 2-week alcohol-free period, both groups consumed 300 mL of red wine for 3 weeks. The blood samples for the iron status analysis were taken at the end of each period. The red wine intake resulted in a decrease in serum hepcidin in both the diabetic subjects (*p* = 0.045) and controls (*p* = 0.001). The levels of serum ferritin also decreased after wine in both groups, reaching statistical significance only in the control subjects (*p* = 0.017). No significant alterations in serum iron, transferrin saturation, or soluble transferrin receptors were found. The suppression of hepcidin, a crucial iron-regulatory hormone and acute-phase protein, in T2D patients and healthy controls, is a novel biological effect of red wine. This may deepen our understanding of the mechanisms of the cardiometabolic effects of wine in T2D.

## 1. Introduction

Diabetes is a growing global health emergency, with a prevalence in the adult population estimated to be 10.5% in 2021 worldwide [1]. Evidence associating iron overload with insulin resistance and type 2 diabetes mellitus (T2D) implies the potential role of hepcidin, the iron-regulatory hormone, in the etiopathogenesis of T2D [2]. This 25-amino-acid peptide is predominantly secreted by hepatocytes, in response to iron burden and inflammation [3,4]. Hepcidin reduces iron bioavailability by triggering the internalization and degradation of ferroportin, the only iron exporter identified so far [5]. When iron efflux from absorptive enterocytes, macrophages, and hepatocytes is repressed, serum iron levels are decreased and intracellular sequestration is enhanced [5,6]. Since iron is essential for living but also potentially toxic, its amount is precisely regulated at both cellular and systemic levels [7]. DNA synthesis and repair, oxygen transport, and cellular respiration are all vital processes demanding iron [8]. However, excess labile cell iron can catalyze the generation of reactive oxygen species (ROS) via the Fenton reaction, causing oxidative tissue damage [9,10]. If pancreatic islet β-cells are affected, insulin secretion could be impaired, and the risk of insulin resistance increased [11,12].

Hepcidin is classified as a type II acute-phase protein [3], with IL-6 being the most dominant inducer of its production [13]. In various inflammatory states, hepcidin is found to be elevated, with the potential to cause anemia or inflammation [6]. Type 2 diabetes is typically associated with chronic low-grade inflammation [14,15], and acute-phase serum protein elevation [14,16]. However, the kinetics of hepcidin in patients with type 2 diabetes remain unclear. Recently published meta-analyses and systematic reviews found no difference [11,17] or only a slight increase in hepcidin in T2D patients compared to controls [18]. However, these inconsistent findings could be partially explained by the use of different and non-standardized assays, as well as a variable consideration of factors that may influence hepcidin expression.

It has been indicated that different food ingredients, including alcohol [19,20,21], might affect hepcidin expression and its levels in serum. On the other hand, the moderate consumption of wine, an important component of the Mediterranean diet [22], has been proven by both observational and interventional trials to reduce cardiometabolic risk and the incidence of type 2 diabetes [23,24,25,26,27]. These beneficial effects have been attributed to both ethanol and polyphenolic compounds [28], which are abundantly present in red wine.

The effect of red wine on serum hepcidin levels in humans is practically unknown. Therefore, the aim of our study was to examine the effects of the moderate consumption of red wine on hepcidin levels in patients with type 2 diabetes mellitus and their non-diabetic controls.

## 2. Materials and Methods

### 2.1. Study Design

Recruitment of participants was conducted through the family medicine practices in the city of Split (Croatia) and within the Department of Endocrinology at the University Hospital of Split during their outpatient visit. Inclusion criteria for both control subjects and patients with type 2 diabetes were the following: (1) males, (2) aged between 40 and 65 years, (3) non-severely obese (BMI < 35 kg/m^2^), (4) non-smokers, and (5) willing to give consent and carry out all study-related procedures.

In addition, control subjects were matched for age and BMI with T2D subjects, in good general health, as determined by screening medical history and clinical examination, and with fasting plasma glucose ≤ 6.9 mmol/L [29]. Diabetic subjects were eligible if they had controlled glycemia (HbA1c value ≤ 7.5% (58 mmol/mol)), received treatment with metformin alone or in combination with other oral antidiabetics.

Both control and diabetic subjects were excluded in cases of: (1) atherosclerotic cardiovascular disease or venous thromboembolism in their medical history, (2) current evidence of acute or chronic inflammatory or infective disease, (3) liver disease, (4) malignancy, (5) dysregulation of iron homeostasis (anemia or hereditary hemochromatosis), (6) previous alcohol or substance abuse, and (7) introduction of new pharmacological agent during the study period.

**Figure 1:**
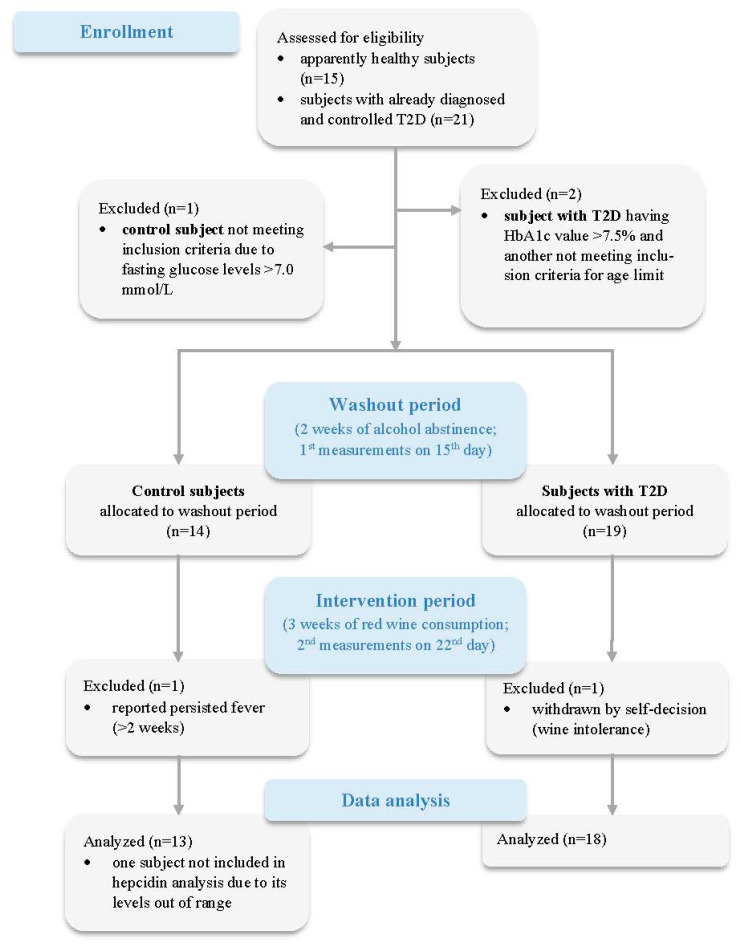
CONSORT flow diagram showing the recruitment processes and study protocol.

After a drive-in period of 2 weeks, in which consumption of any alcoholic beverage was prohibited, subjects in both groups started to drink 300 mL of red wine daily for 3 weeks (Figure 1). This amount had to be split between lunch and dinner and consumed during meals [30]. At the end of the drive-in period, the participants were provided with 9 standard wine bottles of 750 mL. The participants were instructed to maintain their eating and lifestyle habits, including physical activity, during both the drive-in and intervention period. A total of 31 participants completed the study protocol: 18 with type 2 diabetes mellitus and 13 control subjects.

### 2.2. Wine Intervention

Red wine was produced from the Croatian autochthonous red cultivar Plavac mali (*Vitis vinifera* L.) at the Volarević winery, Croatia, 2016. Basic oenological parameters of the used wine are shown in Table S1. Because the composition of phenolic compounds may influence biological effects of wine [31], the results of the analysis of wine used in our study are provided in Table S2.

### 2.3. Anthropometric Assessments and Blood Sampling

At the end of the drive-in and intervention period, participants’ weight, height, and body circumferences were measured. Body mass index (BMI) was calculated as the ratio of weight and the square of height (kg/m^2^). Fasting blood samples were obtained early in the morning and, depending on the type of laboratory parameter, were analyzed the same day or stored at −80 °C for later analysis. In order to prevent the possible influence of diurnal fluctuations in serum hepcidin level, blood collection time was standardized [32]. Three types of vacutainers were used: (1) with K3EDTA, to determine the complete blood count and HbA1c, (2) with fluoride/EDTA, to estimate fasting plasma glucose concentration, and (3) with silica (clot activator)/gel, to separate serum required for the analysis of liver function, inflammation, and serum-based indicators of iron status (UIBC, TIBC, iron, hepcidin, ferritin, soluble transferrin receptors) and glycemic control (fructosamine). Levels of soluble transferrin receptors (sTfR), which reflect the availability of functional iron, were measured using a nephelometric method on a BN ProSpec analyzer (Siemens, ProSpec, Erlangen, Germany). Serum hepcidin was quantified according to the manufacturers’ instructions in a commercially available competitive ELISA kit (Hepcidin 25 (bioactive) HS, DRG Instruments GmbH, Marburg, Germany). All measurements were performed in the Laboratory for Experimental Pharmacology at the University of Split School of Medicine and the Department of Medical Laboratory Diagnostics at the University Hospital of Split.

### 2.4. Statistical Analysis

The data were presented as a mean ± standard deviation (SD) or a median with 95% confidence interval (CI), depending on the data distribution; the normality of distribution was checked using the Shapiro–Wilk test. The significance of differences was assessed using the t-test for normally distributed data or the Mann–Whitney test for deviations from normality. Welch’s correction was used if the assumption of homogeneity of variance was violated. Pearson correlation coefficient was calculated to evaluate the relationship between the BMI value and hepcidin level change. *p*-values less than 0.05 were considered statistically significant. The R programming language for statistical computing version 4.0.2 was used for all statistical analyses.

### 2.5. Ethics Approval

This study complied with the Declaration of Helsinki and its amendments, and was approved by the Ethics Committee of the University of Split School of Medicine, Croatia (no. 2181-198-03-04-13-0042). All subjects gave written informed consent to the sample collection, analysis, and use of the data for publication.

## 3. Results

General characteristics of all participants, along with glucose levels and metabolic parameters related to liver function and the grade of inflammation at the baseline, are shown in Table 1. The mean age of the participants was 52.8 ± 6.3 years, and there was no significant difference between the groups (*p* = 0.075). Subjects with type 2 diabetes and control subjects were also comparable regarding the weight, height, BMI value, waist, and hip circumference.

As expected, T2D subjects showed higher fasting glucose levels in comparison to controls (*p* < 0.0001). Average values of hepatic function parameters, including liver enzymes, albumin, and total bilirubin, were within the reference range in both groups. Similarly, the groups did not differ in hsCRP levels (*p* = 0.317, Table 1).

Hematological and biochemical markers of iron status in the diabetic and control group, at the baseline and post-intervention, are presented in Table 2.

**Figure 2:**
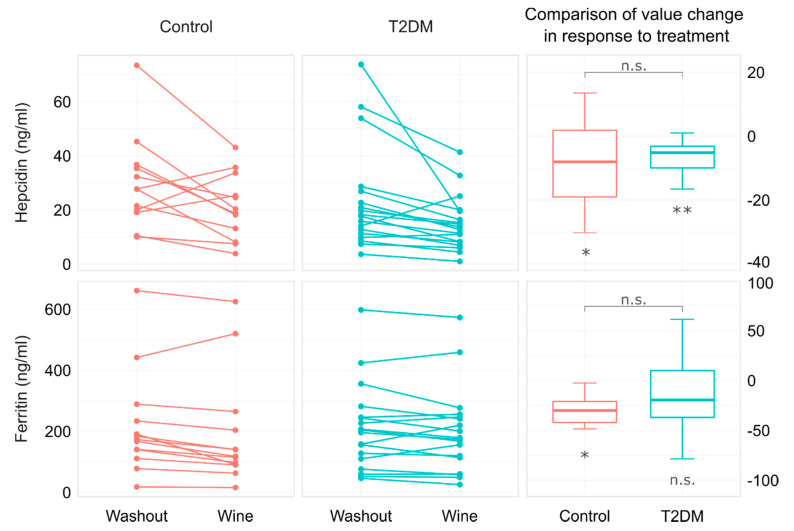
Value changes in hepcidin and ferritin in patients with type 2 diabetes and their age- and BMI-matched controls after 3-week moderate consumption of red wine. The abbreviation n.s. indicates *p* > 0.05, whilst symbols * and ** indicate *p* < 0.05 and *p* < 0.01, respectively.

After 3 weeks of red wine consumption, a significant decrease in hepcidin levels occurred within both groups (*p* = 0.045 and *p* = 0.001 for control and diabetic group, respectively, Table 2, Figure 2). No significant linear relationship between BMI and hepcidin change was observed (Pearson’s r = 0.382, *p* = 0.220 for C; r = 0.037, *p* = 0.883 for diabetic subjects). The decline in serum hepcidin was not mirrored in serum iron, since its levels were not altered following the wine consumption within both groups (*p* = 0.328 and *p* = 0.177 for control and diabetic group, respectively, Table 2). Furthermore, the other standard Fe-related parameters remained largely unchanged after the intervention, except for the ferritin in the control group.

Along with the decrease in hepcidin, a significant decrease in serum ferritin was observed in the control subjects following the red wine intake (*p* = 0.017, Table 2, Figure 2). It should be noted that the hepcidin and ferritin showed the same pattern of change in the diabetic group as well, but in the diabetic patients, the ferritin values around the mean had a wider spread, and statistical significance was not reached.

**Figure 3:**
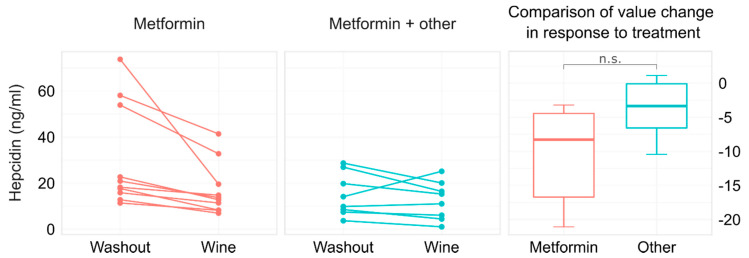
Value changes in hepcidin after 3-week moderate intake of red wine in the patients with type 2 diabetes taking only metformin (*n* = 10) and those taking metformin with other oral antidiabetic agents (*n* = 8). The abbreviation n.s. indicates *p* > 0.05.

Regarding the assessment of glycemic control in the diabetic subjects, neither fasting glucose (7.5 ± 1.4 vs. 7.3 ± 1.4 mmol/L, *p* = 0.294), nor HbA1c values (6.2 (5.9 to 6.7) vs. 6.4 (5.9 to 6.8) %, *p* = 0.322) were affected by the red wine intake. Moreover, the fructosamine levels, which better reflected the average blood glucose concentration over the previous 3 weeks, remained similar before and after intervention (289.0 (270.8 to 294.8) vs. 286.0 (273.0 to 294.8) µmol/L, *p* = 0.524). Glycemic control in the subjects with T2D was achieved with different antidiabetic treatment approaches, which are presented in Table S3. There was no statistical significance in the hepcidin changes in response to red wine consumption between the diabetic patients taking metformin only and those who took metformin in combination with other oral antidiabetics (*p* = 0.062, Figure 3).

## 4. Discussion

The key finding of our study is that a moderate consumption of red wine for 3 weeks is associated with a decrease in serum hepcidin levels in both apparently healthy and in type 2 diabetic patients. To the best of our knowledge, this is the first study to assess the effect of red wine consumption on hepcidin, the iron-regulatory hormone, in human subjects.

The effects of wine on iron status in general have been a matter of discussion. A population-based study showed that there is a dose-response relationship between the chronic daily intake of alcoholic beverages and body iron stores, as determined by serum iron, ferritin, and transferrin saturation (TS) [33]. The decrease in hepcidin levels demonstrated in our study is in line with the findings on the down-regulation of hepcidin expression in in vitro and in vivo models of ethanol ingestion [34,35,36]. As hepcidin falls, ferroportin-mediated iron transport is facilitated, resulting in an increased intestinal iron absorption and iron export from the storage cells. It has been hypothesized that the ethanol-mediated increase in intestinal iron uptake is responsible for elevated body iron indices [19]. However, the suppression of hepcidin levels observed in our study should not be attributed only to the ethanol. Rather, it should be interpreted in the context of the complex chemical composition of wine. Namely, wine phenolics have been shown to inhibit the absorption of dietary iron in the duodenum, presumably due to their iron-chelating ability [37]. Other regulatory pathways might be included as well. For example, the decreased expression of duodenal ferroportin was found in rats treated with quercetin, a well-known flavonoid [38]. In a study with human volunteers, polyphenol-rich red wine, similar to the wine used in our study, was 2- to 3-fold more potent at inhibiting iron absorption than white wine with low polyphenolic content and water [39]. Furthermore, the study indicated that the inhibitory effect on iron absorption could be enhanced if wine is taken with meals [39], which was the method of consumption in our study. Polyphenols were found to form a less soluble complex with iron in the presence of protein-digestion products [39,40]. However, the understanding of the mechanisms of interaction between wine phenolics, ethanol, and iron homeostasis is still limited, and further studies are warranted. The impairment of intestinal iron absorption because of flavonoids and the facilitated export from storage cells due to hepcidin decrease could be an explanation for why changes in serum iron after wine were not found in our study. The other possibility might be that 3 weeks of wine consumption simply was not a sufficiently long period to result in potential changes in iron stores.

Despite the expected release of iron into circulation due to hepcidin decreases [41], we did not find an increase in transferrin saturation. Moreover, the TS in both groups was markedly below 70%, the most frequently considered threshold after which non-transferrin-bound iron (NTBI) could be detected [42]. This iron could induce ROS production and may be implicated in different pathologies, such as vasculotoxicity and atherosclerosis [43]. Hence, the moderate consumption of wine might play a role in maintaining the balance of hepcidin levels needed to prevent iron-induced oxidative stress.

A number of studies indicate that the health-promoting properties of wine in different pathological conditions may include the reduction in inflammation [44,45,46]. When interpreting the concomitant decrease in hepcidin and ferritin observed in our study, it is important to mention that both are regarded as acute-phase proteins, whose levels can rise due to various conditions. Some authors propose that ferritin arises in serum by leaking from damaged cells during inflammation [47]. Serum ferritin can also be elevated due to other pathophysiological components of T2D that are not directly related to iron overload [48], such as insulin resistance [49] and metabolic syndrome [50,51]. In our study, the mean levels of serum ferritin decreased after red wine consumption in both groups, with statistical significance reached only in the controls. This was probably due to the fact that the diabetic group was not large enough to compensate for the wider distribution of the ferritin values observed in the participants with T2D. The parallel decrease in serum hepcidin and ferritin described here is in accordance with other studies demonstrating a linear relationship between serum hepcidin and ferritin [6,41]. The increased export of iron may be associated with decreased ferritin production, which in turn results in decreased circulating ferritin [52]. However, since there is increasing evidence that serum ferritin is not an ideal indicator of body iron status, its serum levels should be cautiously interpreted [48,53,54]. As indicated by the concentration of soluble transferrin receptors (sTfR) [55], the cellular iron demands were unchanged in both groups following the red wine intake. In contrast to ferritin, circulating sTfR is considered a biochemical marker of iron status that is insensitive to inflammation [56].

It has been shown that obesity may also influence hepcidin levels [18,57,58]. Therefore, the participants in our study were matched regarding their BMI values. Our results indicated no correlation between BMI < 35 kg/m^2^ and the hepcidin changes in either the control or the diabetic group. This is in line with the study by Vuppalanchi et al., who showed that elevated hepcidin levels were primarily observed in subjects with BMI ≥ 35 kg/m^2^ [59]. Antidiabetic therapy could also influence the interrelation between iron metabolism and glucose homeostasis [18]. In the studies evaluating hepcidin levels in patients with T2D, information about their antidiabetic therapy is often insufficient [11,17,18]. It has been shown that metformin may suppress hepcidin production [60]. The potential influence of other oral antidiabetic agents on hepcidin levels is practically unexplored. Because we found no difference in hepcidin level changes between the diabetic patients who took metformin alone and those who took metformin in combination with other oral antidiabetics, it is suggestive that these drugs have no opposing effects on hepcidin. The fact that the hepcidin serum levels were also similarly reduced in the non-diabetic control subjects indicates that the hepcidin suppression was primarily mediated by the moderate intake of red wine.

## 5. Conclusions

In conclusion, our study provides experimental evidence of a novel biological effect of moderate red wine consumption that is present in both patients with type 2 diabetes and their apparently healthy controls. The understanding of the effects of red wine on hepcidin, a crucial regulator of iron metabolism and acute-phase protein, may deepen insights into and broaden the understanding of the mechanisms behind the cardiometabolic benefits of the moderate consumption of wine, particularly in diabetic patients. To bring required improvements in diabetes lifestyle guidelines another step closer, further studies assessing the long-term intake of red wine on hepcidin and iron status in larger samples are warranted.

## Figures and Tables

**Table 1:** Anthropometrical and biochemical data of participants with type 2 diabetes mellitus and control subjects at the baseline.

Parameter	Control Group (*n* = 13)	T2D Group (*n* = 18)	*p*-Value
Age (years)	50.5 ± 5.9	54.6 ± 6.2	0.075
Age at T2D onset (years)	N/A	50.6 ± 6.7	N/A
Weight (kg)	100.3 (84.7–105.5)	98.5 (87.2–107.0)	0.617
Height (cm)	186.8 ± 5.6	184.5 ± 10.1	0.427
Waist circumference (cm)	106.0 (95.8–109.5)	107.0 (98.8–112.4)	0.458
Hip circumference (cm)	108.0 ± 5.6	105.0 ± 7.5	0.237
Upper arm circumference (cm)	35.2 ± 2.9	32.2 ± 3.4	0.015
Neck circumference (cm)	42.0 (40.3–44.0)	38.5 (37.0–41.2)	0.051
BMI (kg/m2)	27.2 ± 2.7	29.8 ± 4.1	0.062
Fasting glucose (mmol/L)	5.3 ± 0.4	7.5 ± 1.4	<0.0001
AST (IU/L)	27.1 ± 7.1	22.1 ± 4.9	0.029
ALT (IU/L)	25.0 (19.5–39.2)	25.5 (16.4–29.6)	0.674
GGT (IU/L)	28.0 (23.5–43.7)	25.5 (20.0–32.2)	0.307
Albumin (g/L)	45.0 (45.0–46.0) *	43.0 (41.4–44.0)	0.002
Total bilirubin (µmol/L)	13.8 ± 4.0 †	12.4 ± 4.6	0.388
Urates (µmol/L)	328.9 ± 43.8	359.6 ± 81.2	0.188
hsCRP (mg/L)	1.3 (0.7–1.9)	1.5 (0.8–2.9)	0.317

Abbreviations: AST, aspartate aminotransferase; ALT, alanine aminotransferase; BMI, body mass index; GGT, gamma-glutamyl transferase; hsCRP, high-sensitivity C-reactive protein; N/A, not applicable; T2D, type 2 diabetes. Normally distributed variables are presented as mean ± SD, whilst non-normally distributed variables are presented as median with 95% CIs. * One control participant was excluded from the albumin analysis due to a lack of data for second measurement. † Two control participants were excluded from the total bilirubin analysis due to a lack of data for second measurement.

**Table 2:** Laboratory indicators of the iron status of participants with type 2 diabetes mellitus and control subjects and at pre- and post-intervention.

Laboratory Parameter	Control Group (*n* = 13)			T2D Group (*n* = 18)		
	After Drive-In	After Red Wine	*p*-Value	After Drive-In	After Red Wine	*p*-Value
RBC (×10^12^/L)	5.2 ± 0.5	5.1 ± 0.4	0.077	5.0 ± 0.3	4.9 ± 0.3	0.888
Hematocrit (L/L)	0.45 ± 0.039	0.45 ± 0.034	0.388	0.44 ± 0.019	0.44 ± 0.021	0.749
Hemoglobin (g/L)	154.2 ± 12.6	153.0 ± 10.6	0.348	151.4 ± 6.2	151.5 ± 6.5	0.969
RDW (%)	13.0 ± 0.5	13.2 ± 0.5	0.013	13.5 ± 0.4	13.6 ± 0.5	0.033
MCH (pg)	29.6 ± 1.0	29.9 ± 1.0	0.047	30.7 ± 1.3	30.7 ± 1.4	0.709
MCHC (g/L)	337.5 ± 7.3	339.5 ± 8.8	0.261	341.4 ± 5.5	342.7 ± 6.8	0.349
MCV (fL)	87.1 (84.9–90.1)	87.2 (85.2–91.5)	0.675	89.8 ± 3.6	89.6 ± 3.5	0.495
Serum iron (µmol/L)	21.8 ± 7.1	19.6 ± 6.9	0.328	15.0 (13.8–18.7)	16.4 (14.6–20.9)	0.177
TIBC (µmol/L)	54.2 ± 9.3	53.8 ± 12.1	0.767	57.8 ± 7.2	58.2 ± 7.6	0.434
UIBC (µmol/L)	31.6 ± 11.6	34.2 ± 11.4	0.109	39.4 (37.5–45.5)	41.6 (36.8–46.5)	0.453
Transferrin saturation (%) *	40.6 ± 12.9	37.0 ± 11.3	0.307	26.7 (22.6–29.9)	29.3 (24.5–32.7)	0.265
sTfR (mg/L) ^†^	1.20 ± 0.22	1.24 ± 0.25	0.734	1.05 ± 0.17	1.04 ± 0.20	0.830
Ferritin (ng/mL)	173.0 (126.4–259.8)	118.0 (90.5–232.6)	0.017	209.5 ± 141.5	198.8 ± 139.4	0.215
Hepcidin (ng/mL) ^‡^	30.0 ± 17.3	21.0 ± 12.1	0.045	17.9 (11.9–25.2)	13.2 (8.2–18.3)	0.001

Abbreviations: MCH, mean corpuscular hemoglobin; MCHC, mean corpuscular hemoglobin concentration; MCV, mean corpuscular volume; RDW, red-cell distribution width; RBC, red blood cell; sTFR, soluble transferrin receptor; TIBC, total iron-binding capacity; T2D, type 2 diabetes; UIBC, unsaturated iron-binding capacity. Normally distributed variables are presented as mean ± SD, whilst non-normally distributed variables are presented as median with 95% CIs. * Transferrin saturation percentage calculated as 100 × serum iron/TIBC. ^†^ One control participant had undetectable levels of sTfR, and was thus excluded from the analysis. ^‡^ One control participant had undetectable levels of hepcidin, and was thus excluded from the analysis.

## Data Availability

The data presented in this study are available on request from the corresponding author.

## Supplementary Materials

The following supporting information can be downloaded at: https://www.mdpi.com/article/10.3390/foods11131881/s1. Table S1: Physicochemical properties of red wine Plavac mali. Table S2: Monomeric and oligomeric tannins (mg/L) in Plavac mali. Table S3: Antidiabetic pharmacotherapy used by 18 patients with type 2 diabetes mellitus who completed the study.

## References

[1] (2021). International Diabetes Federation Diabetes Atlas, 10th ed. www.diabetesatlas.org.

[2] Aregbesola A., Voutilainen S., Virtanen J.K., Aregbesola A., Tuomainen T.P. (2015). Serum hepcidin concentrations and type 2 diabetes. World J. Diabetes.

[3] Nemeth E., Valore E.V., Territo M., Schiller G., Lichtenstein A., Ganz T. (2003). Hepcidin, a putative mediator of anemia of inflammation, is a type II acute-phase protein. Blood.

[4] Armitage A.E., Eddowes L.A., Gileadi U., Cole S., Spottiswoode N., Selvakumar T.A., Ho L.P., Townsend A.R., Drakesmith H. (2011). Hepcidin regulation by innate immune and infectious stimuli. Blood.

[5] Nemeth E., Tuttle M.S., Powelson J., Vaughn M.B., Donovan A., Ward D.M.V., Ganz T., Kaplan J. (2004). Hepcidin regulates cellular iron efflux by binding to ferroportin and inducing its internalization. Science.

[6] Ganz T. (2003). Hepcidin, a key regulator of iron metabolism and mediator of anemia of inflammation. Blood.

[7] Camaschella C., Nai A., Silvestri L. (2020). Iron metabolism and iron disorders revisited in the hepcidin era. Haematologica.

[8] Abbaspour N., Hurrell R., Kelishadi R. (2014). Review on iron and its importance for human health. J. Res. Med. Sci..

[9] Kakhlon O., Cabantchik Z.I. (2002). The labile iron pool: Characterization, measurement, and participation in cellular processes. Free Radic. Biol. Med..

[10] Cabantchik Z.I. (2014). Labile iron in cells and body fluids: Physiology, pathology, and pharmacology. Front. Pharmacol..

[11] Liu J.F., Li Q.X., Yang Y.X., Ma L.H. (2020). Iron metabolism and type 2 diabetes mellitus: A meta-analysis and systematic review. J. Diabetes Investig..

[12] Lee H.J., Choi J.S., Lee H.J., Kim W.H., Park S.I., Song J. (2015). Effect of excess iron on oxidative stress and gluconeogenesis through hepcidin during mitochondrial dysfunction. J. Nutr. Biochem..

[13] Wrighting D.M., Andrews N.C. (2006). Interleukin-6 induces hepcidin expression through STAT3. Blood.

[14] Donath M.Y., Shoelson S.E. (2011). Type 2 diabetes as an inflammatory disease. Nat. Rev. Immunol..

[15] Sharif S., Van der Graaf Y., Cramer M.J., Kapelle L.J., de Borst G.J., Visseren F.L.J., Westerink J., SMART Study Group (2021). Low-grade inflammation as a risk factor for cardiovascular events and all-cause mortality in patients with type 2 diabetes. Cardiovasc. Diabetol..

[16] McMillan D.E. (1989). Increased levels of acute-phase serum proteins in diabetes. Metabolism.

[17] Karamzad N., Eftekhari A., Ashrafi-Asgarabad A., Sullman M.J.M., Sahebkar A., Safiri S. (2021). Serum hepcidin, the hepcidin/ferritin ratio and the risk of type 2 diabetes: A systematic review and meta-analysis. Curr. Med. Chem..

[18] Ndevahoma F., Mukesi M., Dludla P.V., Nkambule B.B., Nepolo E.P., Nyambuya T.M. (2021). Body weight and its influence on hepcidin levels in patients with type 2 diabetes: A systematic review and meta-analysis of clinical studies. Heliyon.

[19] Harrison-Findik D.D. (2007). Role of alcohol in the regulation of iron metabolism. World J. Gastroenterol..

[20] Milman N.T. (2020). A review of nutrients and compounds, which promote or inhibit intestinal iron absorption: Making a platform for dietary measures that can reduce iron uptake in patients with genetic haemochromatosis. J. Nutr. Metab..

[21] Omena J., Curioni C., Cople-Rodrigues C.D., Citelli M. (2021). The effect of food and nutrients on iron overload: What do we know so far?. Eur. J. Clin. Nutr..

[22] Eleftheriou D., Benetou V., Trichopoulou A., La Vecchia C., Bamia C. (2018). Mediterranean diet and its components in relation to all-cause mortality: Meta-analysis. Br. J. Nutr..

[23] Martin M.A., Goya L., Ramos S. (2017). Protective effects of tea, red wine and cocoa in diabetes. Evidences from human studies. Food Chem. Toxicol..

[24] Blomster J.I., Zoungas S., Chalmers J., Li Q., Chow C.K., Woodward M., Mancia G., Poulter N., Williams B., Harrap S. (2014). The relationship between alcohol consumption and vascular complications and mortality in individuals with type 2 diabetes. Diabetes Care.

[25] Gepner Y., Golan R., Harman-Boehm I., Henkin Y., Schwarzfuchs D., Shelef I., Durst R., Kovsan J., Bolotin A., Leitersdorf E. (2015). Effects of initiating moderate alcohol intake on cardiometabolic risk in adults with type 2 diabetes: A 2-year randomized, controlled trial. Ann. Intern. Med..

[26] Golan R., Shelef I., Shemesh E., Henkin Y., Schwarzfuchs D., Gepner Y., Harman-Boehm I., Witkow S., Friger M., Chassidim Y. (2017). Effects of initiating moderate wine intake on abdominal adipose tissue in adults with type 2 diabetes: A 2-year randomized controlled trial. Public Health Nutr..

[27] Beulens J.W., van der Schouw Y.T., Bergmann M.M., Rohrmann S., Schulze M.B., Buijsse B., Grobbee D.E., Arriola L., Cauchi S., Tormo M.J. (2012). Alcohol consumption and risk of type 2 diabetes in European men and women: Influence of beverage type and body size The EPIC-InterAct study. J. Intern. Med..

[28] Golan R., Gepner Y., Shai I. (2019). Wine and health—New evidence. Eur. J. Clin. Nutr..

[29] American Diabetes Association Professional Practice Care (2022). Classification and diagnosis of diabetes: Standards of medical care in diabetes—2022. Diabetes Care.

[30] Boban M., Stockley C., Teissedre P.L., Restani P., Fradera U., Stein-Hammer C., Ruf J.C. (2016). Drinking pattern of wine and effects on human health: Why should we drink moderately and with meals?. Food Funct..

[31] Mollica A., Scioli G., Della Valle A., Cichelli A., Novellino E., Bauer M., Kamysz W., Llorent-Martínez E.J., Córdova M.L.F.-D., Castillo-López R. (2021). Phenolic analysis and in vitro biological activity of red wine, pomace and grape seeds oil derived from Vitis vinifera L. cv. Montepulciano d’Abruzzo. Antioxidants.

[32] Busbridge M., Griffiths C., Ashby D., Gale D., Jayantha A., Sanwaiya A., Chapman R.S. (2009). Development of a novel immunoassay for the iron regulatory peptide hepcidin. Brit. J. Biomed. Sci..

[33] Ioannou G.N., Dominitz J.A., Weiss N.S., Heagerty P.J., Kowdley K.V. (2004). The effect of alcohol consumption on the prevalence of iron overload, iron deficiency, and iron deficiency anemia. Gastroenterology.

[34] Harrison-Findik D.D., Schafer D., Klein E., Timchenko N.A., Kulaksiz H., Clemens D., Fein E., Andriopoulos B., Pantopoulos K., Gollan J. (2006). Alcohol metabolism-mediated oxidative stress down-regulates hepcidin transcription and leads to increased duodenal iron transporter expression. J. Biol. Chem..

[35] Harrison-Findik D.D., Klein E., Crist C., Evans J., Timchenko N., Gollan J. (2007). Iron-mediated regulation of liver hepcidin expression in rats and mice is abolished by alcohol. Hepatology.

[36] Ohtake T., Saito H., Hosoki Y., Inoue M., Miyoshi S., Suzuki Y., Fujimoto Y., Kohgo Y. (2007). Hepcidin is down-regulated in alcohol loading. Alcohol. Clin. Exp. Res..

[37] Wang X.M., Li Y., Han L., Li J., Liu C., Sun C.G. (2021). Role of flavonoids in the treatment of iron overload. Front. Cell Dev. Biol..

[38] Lesjak M., Hoque R., Balesaria S., Skinner V., Debnam E.S., Srai S.K.S., Sharp P.A. (2014). Quercetin inhibits intestinal iron absorption and ferroportin transporter expression in vivo and in vitro. PLoS ONE.

[39] Cook J.D., Reddy M.B., Hurrell R.F. (1995). The effect of red and white wines on nonheme-iron absorption in humans. Am. J. Clin. Nutr..

[40] Arfaoui L. (2021). Dietary plant polyphenols: Effects of food processing on their content and bioavailability. Molecules.

[41] Galesloot T.E., Vermeulen S.H., Geurts-Moespot A.J., Klaver S.M., Kroot J.J., Van Tienoven D., Wetzels J.F.M., Kiemeney L.A.L.M., Sweep F.C., Heijer M.D. (2011). Serum hepcidin: Reference ranges and biochemical correlates in the general population. Blood.

[42] Sugiura T., Dohi Y., Takase H., Fujii S., Seo Y., Ohte N. (2021). Analytical evaluation of serum non-transferrin-bound iron and its relationships with oxidative stress and cardiac load in the general population. Medicine.

[43] Vinchi F. (2021). Non-transferrin-bound iron in the spotlight: Novel mechanistic insights into the vasculotoxic and atherosclerotic effect of iron. Antioxid. Redox Signal..

[44] Van Bussel B.C.T., Henry R.M.A., Schalkwijk C.G., Dekker J.M., Nijpels G., Feskens E.J.M., Stehouwer C.D.A. (2018). Alcohol and red wine consumption, but not fruit, vegetables, fish or dairy products, are associated with less endothelial dysfunction and less low-grade inflammation: The Hoorn Study. Eur. J. Nutr..

[45] Imhof A., Woodward M., Doering A., Helbecque N., Loewel H., Amouyel P., Lowe G., Koenig W. (2004). Overall alcohol intake, beer, wine, and systemic markers of inflammation in western Europe: Results from three MONICA samples (Augsburg, Glasgow, Lille). Eur. Heart J..

[46] Li J., Lee D.H., Hu J., Tabung F.K., Li Y., Bhupathiraju S.N., Rimm E.B., Rexrode K.M., Manson J.E., Willett W.C. (2020). Dietary inflammatory potential and risk of cardiovascular disease among men and women in the US. J. Am. Coll. Cardiol..

[47] Kell D.B., Pretorius E. (2014). Serum ferritin is an important inflammatory disease marker, as it is mainly a leakage product from damaged cells. Metallomics.

[48] Adams P.C. (2012). Diabetes: Serum ferritin levels and T2DM—Are body iron stores elevated?. Nat. Rev. Endocrinol..

[49] Pham N.M., Nanri A., Yi S., Kurotani K., Akter S., Foo L.H., Nishi N., Sato M., Hayabuchi H., Mizoue T. (2013). Serum ferritin is associated with markers of insulin resistance in Japanese men but not in women. Metabolism.

[50] Vari I.S., Balkau B., Kettaneh A., André P., Tichet J., Fumeron F., Caces E., Marre M., Grandchamp B., Ducimetière P. (2007). Ferritin and transferrin are associated with metabolic syndrome abnormalities and their change over time in a general population—Data from an Epidemiological Study on the Insulin Resistance Syndrome (DESIR). Diabetes Care.

[51] Park S.K., Kim M.G., Ryoo J.H., Shin J.Y. (2012). Association of serum ferritin and the development of metabolic syndrome in middle-aged Korean men: A 5-year follow-up study. Diabetes Care.

[52] Peng Y.Y., Uprichard J. (2017). Ferritin and iron studies in anaemia and chronic disease. Ann. Clin. Biochem..

[53] Justin C.L., Stevic I., Chan A., Lau K.K.H., Chan H.H.W. (2015). Serum ferritin is not sensitive or specific for the diagnosis of iron deficiency in patients with normocytic anemia. Blood.

[54] Garcia-Casal M.N., Pena-Rosas J.P., Pasricha S.R. (2014). Rethinking ferritin cutoffs for iron deficiency and overload. Lancet Haematol..

[55] Speeckaert M.M., Speeckaert R., Delanghe J.R. (2010). Biological and clinical aspects of soluble transferrin receptor. Crit. Rev. Clin. Lab. Sci..

[56] Skikne B.S., Punnonen K., Caldron P.H., Bennett M.T., Rehu M., Gasior G.H., Chamberlin J.S., Sullivan L.A., Bray K.R., Southwick P.C. (2011). Improved differential diagnosis of anemia of chronic disease and iron deficiency anemia: A prospective multicenter evaluation of soluble transferrin receptor and the sTfR/log ferritin index. Am. J. Hematol..

[57] Andrews M., Soto N., Arredondo-Olguin M. (2015). Association between ferritin and hepcidin levels and inflammatory status in patients with type 2 diabetes mellitus and obesity. Nutrition.

[58] Coimbra S., Catarino C., Santos-Silva A. (2013). The role of adipocytes in the modulation of iron metabolism in obesity. Obes. Rev..

[59] Vuppalanchi R., Troutt J.S., Konrad R.J., Ghabril M., Saxena R., Bell L.N., Kowdley K.V., Chalasani N. (2014). Serum hepcidin levels are associated wih obesity but not liver disease. Obesity.

[60] Hawula Z.J., Wallace D.F., Subramaniam V.N., Rishi G. (2019). Therapeutic advances in regulating the hepcidin/ferroportin axis. Pharmaceuticals.

