# Supplementary material for: Effects of Moderate Consumption of Red Wine on Hepcidin Levels in Patients with Type 2 Diabetes Mellitus
Source: Foods. 2022 Jun 25;11(13):1881. doi: 10.3390/foods11131881 (PMC9266169; doi:10.3390/foods11131881)
Supplement: Supplementary file 1 [file foods-11-01881-s001.zip › foods-1752457-supplementary.pdf]

Table S1. Physicochemical properties of red wine Plavac mali

**Parameter**	**Unit**	**Value**
Density	g/L	998.0
Total ethanol content	%	14.6
Total acids	g/L	3.3
Volatile acids	g/L	0.5
Total dry content	g/L	34.1
Ash	g/L	2.8
pH value	N/A	3.7
CO_2	mg/L	1443.0
Free SO_2	mg/L	23.0
Total SO_2	mg/L	142.0
Total sugar	g/L	21.1
Total phenols	mg GAE/L	2185.75 ± 67.09
Total anthocyanins	mg/L	69.61 ± 3.37

Abbreviation: GAE, gallic acid equivalent.

Table S2. Monomeric and oligomeric tannins [mg/L] in Plavac mali

**Parameter**	**Value**
Catechin	15.98 ± 0.06
Epicatechin	4.01 ± 0.05
Dimer B1	10.89 ± 0.07
Dimer B2	2.65 ± 0.02
Dimer B_3	4.55 ± 0.07
Dimer B_4	2.59 ± 0.005
Trimer C_1	5.63 ± 0.03
Σ monomers	19.99 ± 0.02
Σ dimers	20.68 ± 0.02
Total Flavan-3-ols	46.29 ± 0.03

The results are the mean of two independent measurements and are presented as the arithmetic mean ± SD. (+)-catechin, (-)-epicatechin, procyanidin dimmers B1, B2, B3, B4, and trimer C1, were used as reference compounds for quantitative analysis by HPLC.

Table S3. Antidiabetic pharmacotherapy used by 18 patients with type 2 diabetes mellitus who completed the study

**Antidiabetic treatment**	**Number of participants, n (%)**
Monotherapy with metformin	10 (52.6)
Combination therapy of metformin and:	
α-glucosidase inhibitor	
acarbose	1 (5.3)
DPP-4 inhibitor	
alogliptin	1 (5.3)
sitagliptin	1 (5.3)
vildagliptin	3 (15.8)
Sulfonylurea and DPP-4 inhibitor	
gliclazide and alogliptin	1 (5.3)
SGLT2 inhibitor and DPP-4 inhibitor	
empagliflozine and sitagliptin	1 (5.3)

Abbreviations: DPP-4, dipeptidyl peptidase-4; SGLT2, sodium/glucose cotransporter 2.
